# Food availability aligns with contrasting demographics in populations of an at‐risk songbird

**DOI:** 10.1002/ece3.11557

**Published:** 2024-07-09

**Authors:** Emma C. Keele, Cameron J. Fiss, Darin J. McNeil, Meredith Anderson, Nathan Thomas, Dakotah Shaffer, Jeffery L. Larkin

**Affiliations:** ^1^ Indiana University of Pennsylvania – Research Institute Indiana Pennsylvania USA; ^2^ Department of Environmental and Forest Biology State University of New York College of Environmental Science and Forestry Syracuse New York USA; ^3^ Department of Forestry and Natural Resources University of Kentucky Lexington Kentucky USA; ^4^ Ecology and Evolutionary Biology Interdisciplinary Program, School of Veterinary Medicine and Biomedical Sciences Texas A&M University College Station Texas USA; ^5^ Department of Biology Shippensburg University Shippensburg Pennsylvania USA; ^6^ Department of Biology Indiana University of Pennsylvania Indiana Pennsylvania USA; ^7^ American Bird Conservancy The Plains Virginia USA

**Keywords:** food availability, Golden‐winged warbler, leaf‐roller caterpillar, plasma lipid metabolites

## Abstract

Golden‐winged Warblers (*Vermivora chrysoptera*) have become rare across much of their historic breeding range and response to conservation efforts is variable. Evidence from several recent studies suggests that breeding output is a primary driver explaining responses to conservation and it is hypothesized that differences in food availability may be driving breeding output disparity between two subpopulations of the warbler's Appalachian breeding range. Herein, we studied two subpopulations: central Pennsylvania (“central subpopulation”), where breeding productivity is relatively low, and eastern Pennsylvania (“eastern subpopulation”), where breeding productivity is relatively high. To test the food‐availability hypothesis in this system, we measured density of caterpillars, plasma lipid metabolites (triglycerides [TRIG; fat deposition] and glycerol [GLYC; fat breakdown]), body mass of adults males, and acquired body mass data for fledglings at 38 sites managed for nesting habitat. Consistent with our prediction, leaf‐roller caterpillar density, the group upon which Golden‐winged Warblers specialize, was 45× lower in the central subpopulation than the eastern subpopulation. TRIG concentrations were highest within the eastern subpopulation during breeding grounds arrival. The change in TRIG concentrations from the breeding‐grounds‐arrival stage to the nestling‐rearing stage was subpopulation dependent: TRIG decreased in the eastern subpopulation and was constant in the central subpopulation, resulting in similar concentrations during the nestling‐rearing stage. Furthermore, GLYC concentrations were higher in the eastern subpopulation, which suggests greater energy demands in this region. Despite this, adult male warblers in the eastern subpopulation maintained a higher average body mass. Finally, fledgling body mass was 16% greater in the eastern subpopulation than the central subpopulation before and after fledging. Collectively, our results suggest that poor breeding success of Golden‐winged Warblers in the central subpopulation could be driven by lower availability of primary prey during the breeding season (leaf‐roller caterpillars), and this, in turn, limits their response to conservation efforts.

## Graphical abstract

Herein, we studied two subpopulations: central Pennsylvania (“central subpopulation”), where breeding productivity is relatively low, and eastern Pennsylvania (“eastern subpopulation”), where breeding productivity is relatively high. To test the food‐limitation hypothesis in this system, we measured density of caterpillars, plasma lipid metabolites (triglycerides [TRIG; fat anabolism] and glycerol [GLYC; fat catabolism]), body mass of adults, and acquired body mass data for fledglings at 38 sites managed for nesting habitat. Collectively, our results suggest that poor breeding success of Golden‐winged Warblers in the central subpopulation could be driven by a limitation of primary prey during the breeding season (leaf‐roller caterpillars), and this, in turn, limits their response to conservation efforts.

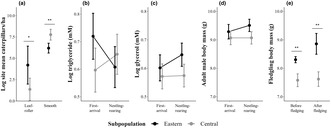

## 1 INTRODUCTION

Effective wildlife conservation relies on linking species' demographic rates to influential environmental factors (Johnson, 2007). For songbirds, researchers often use estimates of breeding productivity (i.e., nest success, fledgling survival, productivity, etc.) to predict the viability of a given population (Gjerdrum et al., 2005; Matthews et al., 2013; Stillman et al., 2019). These demographic rates are often linked with local habitat metrics (e.g., vegetation structure and composition) and these relationships are subsequently translated into practical habitat management recommendations (Boves et al., 2013; Patterson & Best, 1996; Terhune II et al., 2016). Although, populations may not respond to conservation efforts if factors driving their demographic rates (e.g., predation, competition, and food availability) are not closely aligned with local habitat metrics (e.g., vegetation structure and composition [Winter et al., 2005]) or are not explicitly addressed in habitat management recommendations.

Reduced food availability can negatively influence avian reproductive success and fecundity (Duguay, 1997; Lack, 1968; Martin, 1987), and, thus, can act as an important factor in regulating populations. For example, this has been shown to constrain reproduction at many stages of the avian breeding cycle including clutch size (Seress et al., 2018), nestling growth and survival (Martay et al., 2023), re‐nesting and double‐brooding (Nagy & Holmes, 2005), and post‐fledging survival (Wiens et al., 2006). Gauging the effects of prey availability on demographic rates of insectivorous bird populations is often achieved by measuring prey abundance (Rodenhouse & Holmes, 1992), although lower prey abundance alone does not always explain avian population trajectories (Owen et al., 2005; Williams et al., 2007). To elucidate some of these complex relationships, researchers have quantified physiological response of individuals to variable levels of prey by measuring plasma lipid metabolites, stress hormones, or body condition indices (Albano, 2012).

Plasma lipid metabolites can be useful to assess the effects of differences in food availability because these metabolites are short‐term predictors of a bird's metabolic state (feeding/fasting within 2–8 h of blood collection [Jenni‐Eiermann & Jenni, 1994; Smith et al., 2007; Zajac et al., 2006]). Two commonly measured plasma lipid metabolites are triglycerides (hereafter “TRIG”), in which higher concentrations are indicative of fat anabolism (i.e., deposition) from recent periods of feeding and which is positively associated with daily mass gain, and glycerol (hereafter “GLYC”), in which higher concentrations are indicative of fat catabolism (i.e., breakdown) from recent periods of fasting/exercise and which is positively associated with daily mass loss (Guglielmo et al., 2005; Jenni‐Eiermann & Jenni, 1994; Smith et al., 2021). When interpreting plasma lipid metabolite results it is important to consider life stage and sex, given that males and females exhibit distinct physiological responses during different times of the year (e.g., migration and breeding [Jenni‐Eiermann & Jenni, 1994; Kern et al., 2005]). For example, higher rates of fat catabolism (e.g., elevated GLYC concentrations) during migratory stopover often suggest the individual has undergone fasting as a consequence of poorer stopover habitat quality (Guglielmo et al., 2005; Smith et al., 2021). Whereas, during breeding relatively high GLYC concentrations has been reported to be associated with greater energy demands, particularly for males (e.g., increased nestling provisioning [Kern et al., 2005; Owen et al., 2005]), and higher energy demands have in turn been linked to greater reproductive success (Done et al., 2011). Assessing plasma lipid metabolites coupled with prey availability provides an effective way for researchers to compare variable food resources and the avian energetic response across different breeding stages in populations (Albano, 2012).

The Golden‐winged Warbler (*Vermivora chrysoptera*) is a Neotropical‐Nearctic migratory songbird that nests within early‐successional communities in forested regions of eastern North America (Confer et al., 2020). Due to this species' steep population decline (−1.8% year^−1^ range‐wide from 1966 to 2021 [Sauer et al., 2021]), the species is now being considered for listing under the US Endangered Species Act (Sewell, 2010). The Golden‐winged Warbler is a case example for how even if best management practices are followed to create high‐quality nesting habitat (Roth et al., 2019), a positive population response may not be observed if other threats exist (e.g., predation, competition, and food availability). For example, even though nest site vegetation structure and composition matched Golden‐winged Warbler preferences within study sites in New York, competition with Blue‐winged Warblers (*Vermivora cyanoptera*) and Brown‐headed Cowbirds (*Molothrus ater*) reduced clutch sizes (Confer et al., 2003). Moreover, the assessment of federal programs focused on creating Golden‐winged Warbler nesting habitat found predicted warbler density and occupancy to be dependent upon breeding output [independent juveniles produced/pair/year], especially in Appalachia (McNeil, Rodewald, Robinson, et al., 2020; McNeil, Rodewald, Ruiz‐Gutierrez, et al., 2020). Variable responses to conservation efforts throughout the breeding range suggest Golden‐winged Warblers' reproductive success may be influenced by factors that are not currently addressed in management practices (i.e., prey availability).

A disparity in prey availability has been hypothesized to be a potential driver of starkly divergent Golden‐winged Warbler population trends between central Pennsylvania (hereafter “central subpopulation”) and eastern Pennsylvania (hereafter “eastern subpopulation” [McNeil et al., 2024]). Within managed Golden‐winged Warbler nesting habitat, the eastern subpopulation is documented to be more productive in many respects; this subpopulation is reported to have 10% greater clutch sizes, 9% heavier nestlings, 35% more fledglings produced per successful nest, 184% (~3×) greater full‐season productivity, 100% (2×) lower begging by fledglings, and 177% (~3×) greater likelihood of adult site occupancy than the central subpopulation (Larkin & Bakermans, 2012; McNeil et al., 2024; McNeil, Rodewald, Robinson, et al., 2020). A food‐availability hypothesis has been suggested (McNeil et al., 2024) given that other factors have not been found to influence Golden‐winged Warbler reproductive success; such as, warblers from the central and eastern subpopulations overwinter together (Kramer et al., 2018) and vegetation structure did not predict warbler nest survival within managed Appalachian nesting sites (including Pennsylvania [McNeil et al., 2017]).

In this study, we tested the hypothesis that the central Golden‐winged Warbler subpopulation, where breeding output and responses to conservation action are weak, had lower food availability than the eastern subpopulation, where both patterns are reversed (McNeil et al., 2024; McNeil, Rodewald, Robinson, et al., 2020; McNeil, Rodewald, Ruiz‐Gutierrez, et al., 2020). To test this hypothesis, we compared primary prey availability (leaf‐roller caterpillars; family: Tortricidae) and physical indicators of food availability (plasma lipid metabolites, body mass from adult males, and fledgling body mass) between the two subpopulations. We predicted that: (1) leaf‐roller caterpillar density would be lower in the central subpopulation, (2) adult male Golden‐winged Warbler TRIG concentrations would be lower in the central subpopulation at the breeding‐grounds‐arrival and nestling‐rearing stages, (3) adult male warbler GLYC concentrations would be higher in the central subpopulation at the breeding‐grounds‐arrival and nestling‐rearing stages, (4) adult male warbler body mass would be equal upon breeding grounds arrival and body mass would be lower in the central subpopulation during the nestling‐rearing stage, and (5) fledgling warbler body mass would be lower in the central subpopulation. An assessment of food availability between these two breeding subpopulations in Pennsylvania may have implications for other portions of the Golden‐winged Warblers' breeding range and for future research and management.

## 2 METHODS

### 2.1 Study area

**FIGURE 1:**
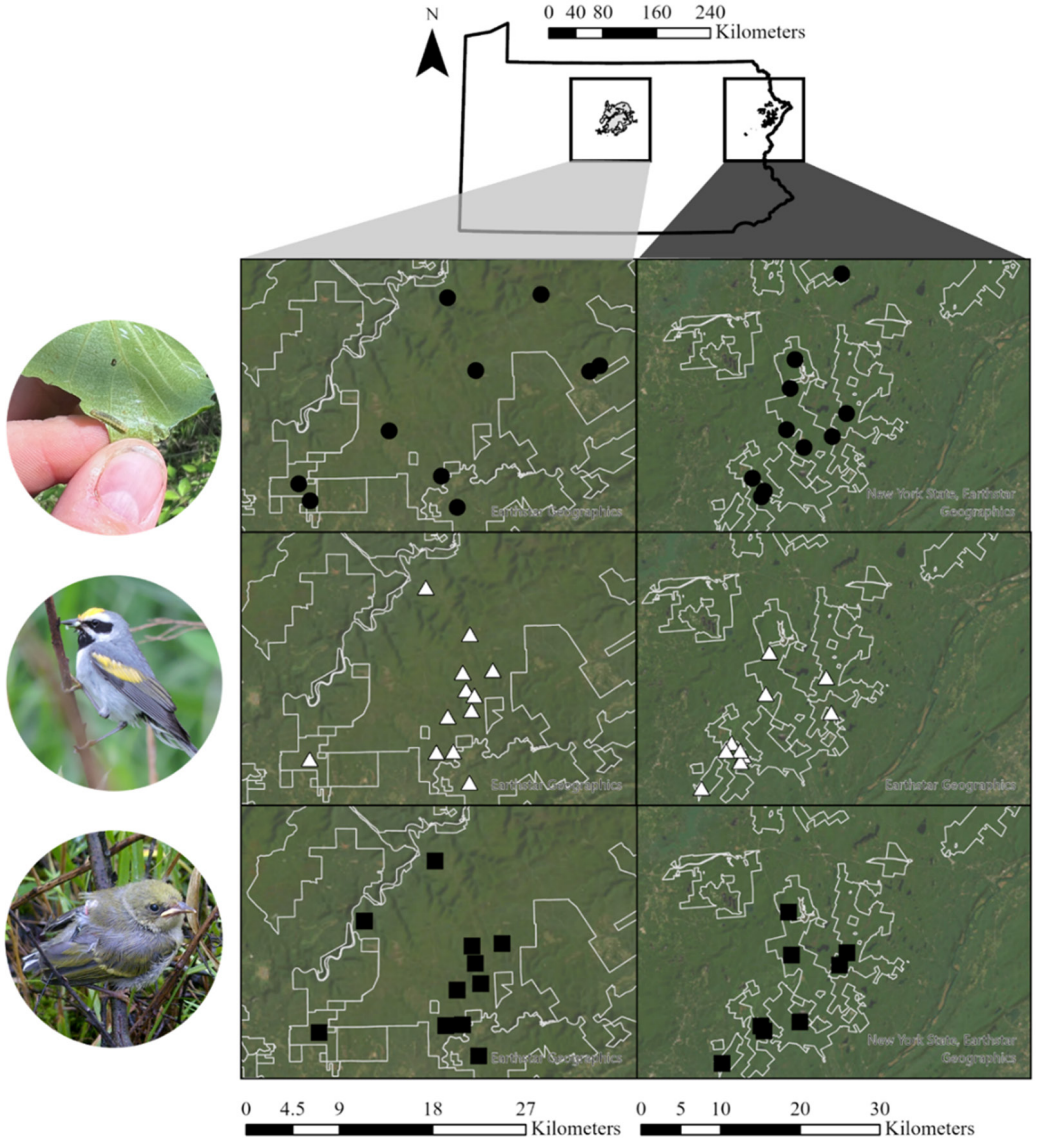
Sampled managed nesting sites in the Golden‐winged Warbler central subpopulation (left panels) and eastern subpopulation (right panels) in Pennsylvania. Across 38 managed nesting sites, we measured caterpillar density at 20 sites in 2018 (first row), collected blood samples and body mass from adult male warblers at 22 sites in 2018 (second row), and acquired body mass of fledgling warblers at 19 sites in 2014–2017 (third row; McNeil et al., 2024). Sampling effort overlapped at some sites (all: 5, caterpillar and adult males: 4, fledgling and adult males: 9, only adult males: 4, only caterpillars: 11, only fledglings: 5).

We surveyed 38 sites (mean: 50.78 ha, range: 1.07–311.24 ha) managed as Golden‐winged Warbler nesting habitat (Figure 1), which included recent timber harvests (≤10 years since past timber harvest) and a managed utility right‐of‐way. The boundary of our study sites aligned with the perimeter of the timber harvest (or other early‐successional wood patch) and we received this information from shapefiles created by the Pennsylvania Game Commission and the Pennsylvania Department of Conservation and Natural Resources. Landscape and within‐site features that characterized managed Golden‐winged Warbler nesting sites were: deciduous‐forest‐dominated landscape (>70%), regenerating hardwood stands with 2.3–9.2 m^2^/ha residual basal area, and high structural complexity and the interspersion of herbaceous vegetation (e.g., grass and broadleaf plants [Bakermans et al., 2015; McNeil et al., 2023; Roth et al., 2014, 2019]). Competition/hybridization with Blue‐winged Warblers was expected to be minimal across both subpopulations because naïve occupancy of this species is reported to be low in our study areas (6%–7% [McNeil, Rodewald, Robinson, et al., 2020]).

In the central subpopulation, we sampled within Sproul State Forest and State Game Lands 100 (Centre and Clinton counties; Figure 1). The central subpopulation was within the Deep Valley section of the Appalachian Plateau physiographic province and the range in elevation is 500–750 m (Shultz, 1999). The dominant forest type was oak‐hickory (Fike, 1999). Within managed warbler nesting sites in this subpopulation, the most common sapling species were aspens (*Populus* spp.), black birch (*Betula lenta*), black cherry (*Prunus serotina*), pin cherry (*P*. *pennsylvanica*), pines (*Pinus* spp.), red maple (*Acer rubrum*), and oaks (*Quercus* spp.). Common shrub species included black huckleberry (*Gaylusaccia baccata*), blueberries (*Vaccinium* spp.), mountain laurel (*Kalmia latifolia*), blackberry (*Rubus* spp.), and witch hazel (*Hamamelis virginiana* [Fiss et al., 2020]).

In the eastern subpopulation, we sampled within Delaware State Forest (Pike and Monroe counties; Figure 1). The eastern subpopulation was within the Glaciated Low Plateau section of the Appalachian Plateau and the range in elevation is 300–600 m (Shultz, 1999). Like the central subpopulation, oak‐hickory was the dominant forest type (Fike, 1999), but, in contrast to the central subpopulation, this region had a higher abundance of natural wetlands (Davis, 1993). The most common sapling species recorded within Golden‐winged Warbler nesting habitat in this subpopulation were aspens, black birch, black cherry, hickories (*Carya* spp.), serviceberries (*Amelanchier* spp.), and oaks. Common shrubs included scrub oak (*Q*. *ilicifolia*), blueberries, black huckleberry, and witch hazel (Fiss et al., 2020). More information about these two subpopulations can be found in Fiss et al. (2020, 2021), McNeil, Rodewald, Robinson, et al. (2020), and McNeil, Rodewald, Ruiz‐Gutierrez, et al. (2020).

### 2.2 Caterpillar density

**FIGURE A1:**
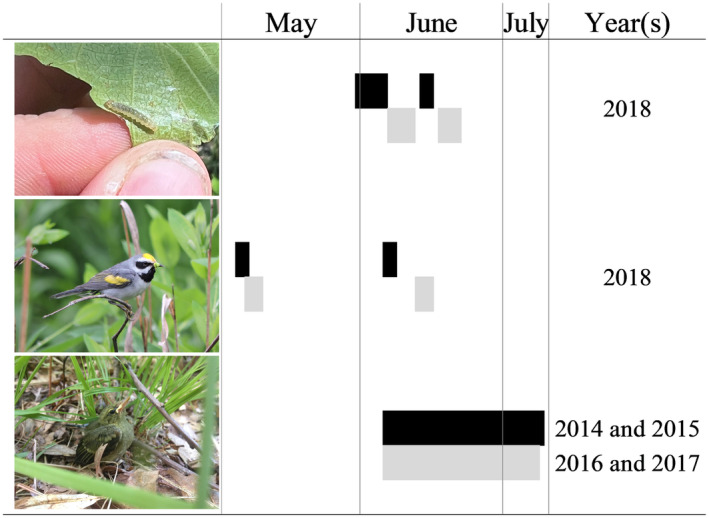
Timing of sampling effort for data collection for caterpillar density, blood/body mass from adult male Golden‐winged Warblers, and body mass from fledglings. Sampling began on 4 May and continued through 9 July in the eastern (black) and central (gray) subpopulations in Pennsylvania. Caterpillar density was sampled twice in 2018 (eastern: 30 May–5 June and 13–15 June, central: 06–11 June and 17–21 June). Blood and body mass from adult male warblers were sampled twice in 2018 (eastern: 4–6 May and 5–7 June, central: 6–9 May and 12–15 June). Body mass from fledgling warblers was sampled in 2014 and 2015 in the eastern subpopulation (5 June–9 July) and in 2016 and 2017 in the central subpopulation (5 June–8 July; McNeil et al., 2024).

**FIGURE A2:**
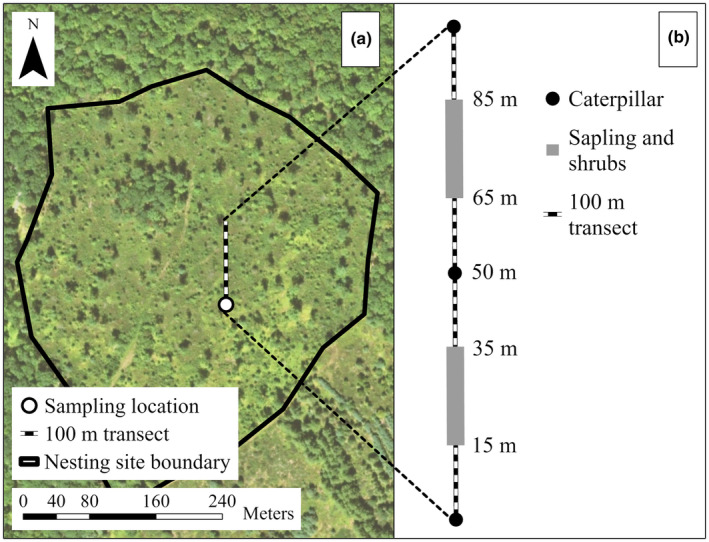
Caterpillars were sampled from 12 sapling and shrub focal species twice during the summer of 2018 at 10 Golden‐winged Warbler managed nesting sites within the two subpopulations in Pennsylvania. A random sampling location starting point was identified within each site, and a random bearing was chosen to create a 100‐m transect (a). Along this 100‐m transect, caterpillars were sampled at three locations (0, 50, and 100 m) and 12 sapling and shrub focal species were sampled within two (2 m × 20 m) plots (b).

To measure prey availability, we sampled caterpillars within 10 managed nesting sites in each subpopulation (20 total sites). We visited each site twice in 2018. Because Golden‐winged Warbler activity is known to occur several days sooner in the eastern subpopulation than the central subpopulation (McNeil, 2019), we sampled caterpillars in the eastern subpopulation first during each visit (eastern: 30 May–5 June and 13–15 June, central: 6–11 June and 17–21 June, Figure A1). We choose these dates because they corresponded to when adult Golden‐winged Warblers are known to provision their young in both subpopulations (McNeil, 2019). To place random points within each site, we used the Create Random Points tool from ArcGIS 10.3 (ESRI, Redlands, California, USA). From this point, we randomly selected a compass bearing (degrees) and delineated a 100‐m transect. If the 100 m transect extended beyond the managed site boundary, we selected an alternate bearing at random (Figure A2). At three locations along the 100 m transect (0, 50, and 100 m), we searched for the nearest individual of each of 12 focal sapling and shrub species within a 50‐m radius. These 12 species were: blackberry, black birch, black cherry, chestnut oak (*Q*. *montana*), mountain laurel, pin cherry, red maple, red oak (*Q*. *rubra*), serviceberry, scrub oak, white oak (*Q*. *alba*), and witch hazel. We selected these species because Golden‐winged Warblers are known to preferentially forage on these species for prey (Bellush et al., 2016; Ficken & Ficken, 1968). When a focal plant was found, we placed a 1‐m^2^ white sheet on the ground beneath its foliage and struck its base with a rubber mallet and shook it vigorously three times to capture caterpillars (Cooper & Whitmore, 1990). After we attempted to dislodge caterpillars with the hammer and shaking, we also manually searched every leaf for leaf‐roller caterpillars. We gently bent tall saplings, ≤3.5 m, to reach the canopies for manual searching. To minimize variation in the size of focal plants searched for caterpillars, we only selected saplings <10 cm diameter at breast height and 0.5–3.5 m tall and shrubs >0.5 m tall. Additionally, we measured the height (m) and crown width (m) of each focal plant sampled for caterpillars to estimate focal plant area (m^2^) as a proxy for leaf area or biomass (Cooper & Whitmore, 1990; Marshall et al., 2002). In the field, we categorized caterpillars into two morphogroups: leaf‐rollers (found tightly rolled within a leaf, Torticidae) and smooth (not rolled within a leaf and no hair‐like setae present). Our smooth caterpillar category may have included a combination of leaf‐rollers and smooth caterpillar species because not all leaf‐rollers roll leaves, not all leaf‐roller instars roll leaves (Gilligan, 2003), and some leaf‐rollers may have fallen from their rolled leaves. We also counted bristled caterpillars (hair‐like setae present) but did not include this morphogroup because these structures are used as a defense against bird predation (Lichter‐Marck et al., 2015), so they are likely not a prey item for Golden‐winged Warblers. Lastly, we categorized caterpillars into two size classes, <3 and >3 cm. Although Golden‐winged Warblers are known to eat a variety of invertebrates (e.g., spiders [Jacobs, 1904] and ants [Streby et al., 2014]), caterpillars are the main component of their diet during the breeding season (Confer et al., 2020; Will, 1986) and they are thought to specialize on leaf‐rolling species (e.g., Tortricidae, *Pandemis* spp., *Episimus* spp. [Will, 1986]). In Minnesota and Manitoba, Canada, the mass from stomach contents from Golden‐winged Warbler nestlings and young fledglings were found to be 89% leaf‐roller caterpillars (*Archips* spp.) and adults were observed almost always bringing back leaf‐roller caterpillars to chicks in video‐monitored nests (Streby et al., 2014). During each visit, we recorded the number of caterpillars belonging to each morphogroup on each focal plant searched (number of individuals/stem).

To estimate density of the 12 focal sapling and shrub species at each managed nesting site, we counted the number of stems per species within two 20 m × 2 m (40 m^2^) plots (at 15–35 and 65–85 m along the same 100 m transect; Figure A2). We counted saplings (<10 cm diameter at breast height and 0.5–3.5 m tall) and shrubs (>0.5 m tall) that originated from the ground within the sample plot. We distributed the sampling of caterpillars and woody stem density along the 100 m transect to cover a greater area of the managed nesting site. For each visit, we calculated the average absolute density of each caterpillar morphogroup (caterpillars/ha) by multiplying the number of caterpillars per stem (individuals/stem) by the number of woody stems per hectare (stems/ha) for each focal sapling and shrub species. Then, we averaged the two visits to acquire site mean caterpillars/ha. We calculated the percent change ([(final − initial)/initial] × 100%) for each caterpillar morphogroup to quantify the degree to which the subpopulations differed.

### 2.3 Adult male blood and body mass

To assess physical indicators of variable food resources in adult male Golden‐winged Warblers, we measured physiological and morphological conditions of individual birds. In 2018, we sampled warblers from 12 and 10 managed nesting sites in the central and eastern subpopulations, respectively (22 total). Importantly, we sampled each site twice, corresponding with two distinct phases of the annual breeding cycle: (1) when males arrived at breeding grounds and initiated territories, hereafter “first‐arrival” (eastern: 4–6 May, central: 6–9 May, Figure A1) and (2) when males and females both provision nestlings (Fiss et al., 2016; McNeil, 2019), hereafter “nestling‐rearing” (eastern: 5–7 June, central: 12–15 June, Figure A1). We selected these dates based on extensive prior work conducted within both subpopulations (Fiss et al., 2020, 2021; McNeil et al., 2024; McNeil, Rodewald, Robinson, et al., 2020; McNeil, Rodewald, Ruiz‐Gutierrez, et al., 2020). Because both stages are known to occur earlier in the eastern subpopulation (McNeil, 2019), we always sampled this region first. We used 6–12 m mist nets (30 mm mesh size; Avinet, Portland, ME) and conspecific playback to capture males from 0530 to 1400 (start time–end time). We recorded the number of seconds required to obtain the blood sample from individuals. We collected all blood samples within 3.5 min of capture to reduce handling‐induced variations in plasma lipid metabolite concentrations that can occur with longer bleed times (Guglielmo et al., 2002). We collected no more than 1% of the bird's body weight (g) in blood from the brachial vein using a 27‐gauge needle and heparinized capillary tubes (Fair & Jones, 2010; Owen, 2011). Immediately after blood collection, we stored samples in a small cooler with ice packs and once we returned from the field at the end of each day (samples were in cooler with ice packs for approximately 4 h), we centrifuged the blood and stored the plasma and red blood cells separately at approximately −18°C until analysis. We collected blood samples to measure triglyceride (TRIG) and glycerol (GLYC) concentrations. For each bird we also recorded body mass with a digital scale (0.01 g accuracy), wing chord (mm), and age (after‐hatch‐year [AHY], second‐year [SY], and after‐second‐year [ASY]) by assessing molt (Pyle et al., 1997).

### 2.4 Fledgling body mass

We acquired Golden‐winged Warbler fledgling body mass data from McNeil et al. (2024). We sampled fledglings for 2 years in each subpopulation (eastern: 2014–2015 and central: 2016–2017) and from 8 and 11 managed nesting sites in the eastern and central subpopulations, respectively (19 total; Figure A1). We weighed fledglings from monitored nests or if found opportunistically while walking through the managed nesting sites. Most often on day eight post‐hatching (unless individuals fledged sooner), we gently placed all fledglings from each brood into a cloth bag and without looking, randomly selected two individuals to weigh (g). Lastly, we record the age (number of days since hatching) for individuals from monitored nests. For fledglings captured opportunistically, we estimated age based on plumage condition.

### 2.5 Plasma lipid metabolites

To measure TRIG and GLYC concentrations from adult male plasma samples, we followed procedure (A) from the Sigma Serum Triglyceride Determination Kit (Catalog Number TR0100, Sigma Chemical, St. Louis, Missouri). We used a Gensys 5 Spectrophotometer (ThermoSpectronic, model: 336001) and ran assays in 1.5 mL polystyrene UV/Vis semi‐micro cuvets (United Laboratory Plastics, St. Louis, Missouri). To summarize the procedure, first, we prepared the free GLYC and TRIG reagents and set the spectrophotometer wavelength to 540 nm. Next, we pipetted the instructed volumes of free GLYC reagent into each cuvet followed by water, GLYC standard, or plasma samples in cuvets respectively labeled blank, standard, and sample. After incubation (15 min), we used a spectrophotometer to read the initial absorbance values. Finally, we added the TRIG reagent to each cuvet and re‐ran samples to read the final absorbance values. Using the initial and final absorbance readings we followed the instructions to calculate the concentrations (mM) of free GLYC, true TRIG, and total TRIG. For all following analyses, we used concentrations of free GLYC and true TRIG. When possible, we duplicated individual plasma samples and averaged their values (*n* = 43, 52%). We were not able to run duplicates for individuals that did not have enough blood collected (*n* = 39, 48%).

### 2.6 Statistical analyses

We used the program R version 4.1.1 for all statistical analyses (R Core Team, 2021). To compare site average caterpillar density (leaf‐roller and smooth morphogroups run separately), site average woody stem density (12 focal species run separately), focal plant area (12 focal plant species pooled), and witch hazel plant area between subpopulations, we used two‐tailed *t*‐tests (*t*.*test* function, *stats* package). We tested witch hazel plant area separately because of the 12 focal plant species 94% of the leaf‐roller caterpillars we counted were on witch hazel and leaf‐roller caterpillars are Golden‐winged Warblers' primary prey (Streby et al., 2014). Prior to analyses, we log‐transformed caterpillar densities, woody stem densities, and TRIG and GLYC concentrations due to non‐normality and after log‐transformation these response variables approximated normality. To test our prediction if TRIG, GLYC, and adult male body mass were influenced by an interaction term and the main effects (subpopulation × breeding stage), while accounting for other variables, we used backwards stepwise multiple regression methods (as in Guglielmo et al., 2005). Our two main effects were subpopulation (central and eastern) and breeding stage (first‐arrival and nestling‐rearing), and our other variables were scaled mass index (SMI; index for body condition), bleed time, wing chord, and age. We found that adult male body mass and wing chord were strongly correlated (Pearson's correlation coefficient, *cor*.*test* function, *stats* package; *t* = 1.97, *df* = 80, *p* = .05, *ρ* = .22); therefore, we calculated SMI by regressing wing length onto body mass following Peig and Green (2009). Bleed time was the time from when an individual bird was captured to when the blood drawing was finished. All continuous variables were scaled to have a mean of 0 and standard deviation of 1 using the *scale* function. The explanatory variables in our TRIG and GLYC global models were the interaction term and main effects, age, bleed time, and SMI. The explanatory variables in our adult male body mass global model were the interaction term and main effects, age, and wing chord. During our backwards selection process, first, we tested for effects of a small set of variables (age, bleed time, and SMI or wing chord) by stopping when *p* < .10. Next, we tested our prediction by assessing the interaction term. If the interaction term was not influential (*p* > .10), we removed this and continued to test the main effects. Our final model for each response variable was determined by all explanatory variable's *p* < .10 (Guglielmo et al., 2005) and *β* 90% confidence intervals (CI) not overlapping zero. Lastly, to assess if average fledgling body mass (McNeil et al., 2024) differed between subpopulations, we ran two separate two‐tailed *t*‐tests for individuals weighed before fledging and for individuals weighed after fledging. We tested the two age groups separately because fledgling body mass was correlated with age in both subpopulations (Pearson's correlation coefficient; central: *t* = 4.79, *df* = 64, *p* < .01, *ρ* = .34 and eastern: *t* = 5.16, *df* = 88, *p* < .01, *ρ* = .48). Additionally, we ran four more separate two‐tailed *t*‐tests to assess if fledgling body mass varied between the two sampling years within each subpopulation for the two age groups.

## 3 RESULTS

### 3.1 Caterpillar density

**FIGURE 2:**
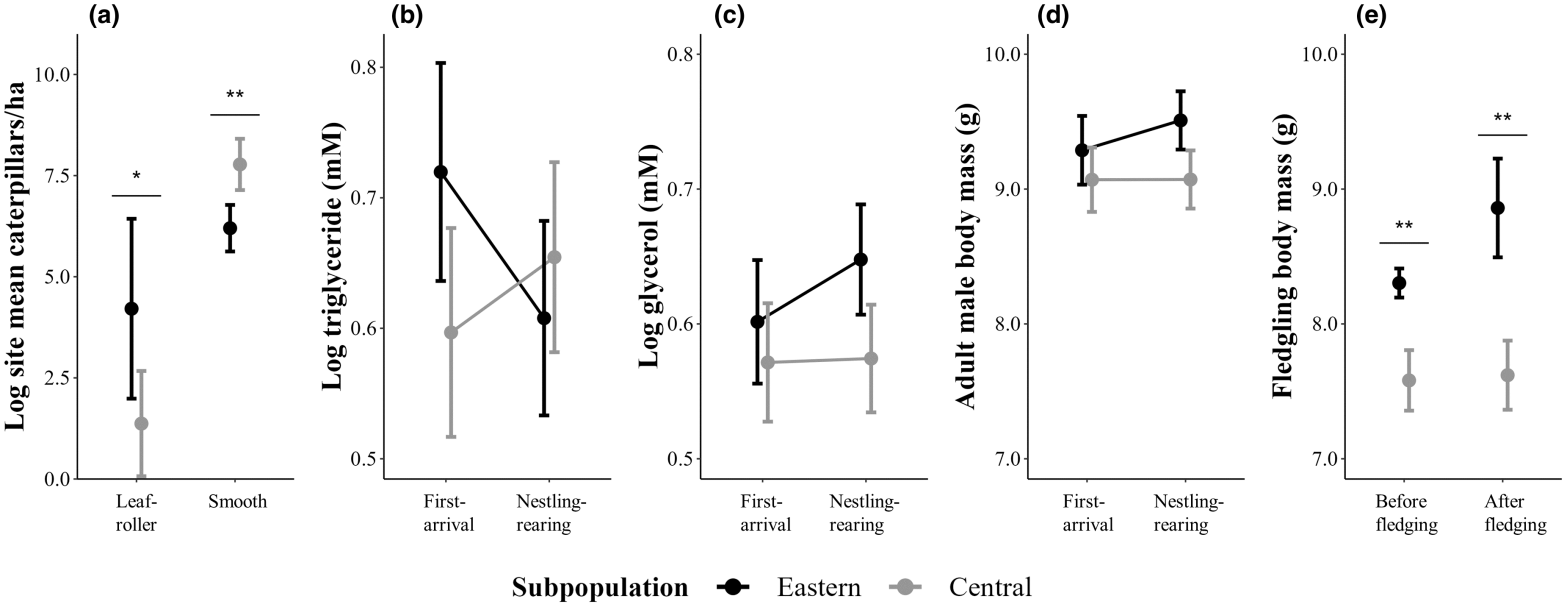
Comparison of log‐transformed caterpillar densities between the Golden‐winged Warbler eastern and central subpopulations in Pennsylvania (a). Log‐transformed triglyceride concentrations (b), log‐transformed glycerol concentrations (c), and adult male body mass (d) were assessed with backwards stepwise multiple regression to test our predictions of an interaction term and the main effects (subpopulation × breeding stage). Comparison of fledgling body mass between subpopulations weighed before and after fledging (e). Error bars represent 95% confidence intervals and statistical differences from two‐tailed *t*‐tests are denoted with *p ≤ .05 and **p ≤ .01.

We counted 818 individual caterpillars (leaf‐roller = 90, smooth = 728) during 40 site visits in 2018. All leaf‐roller caterpillars were <3 cm and most smooth caterpillars were as well (<3 cm = 721, >3 cm = 7); therefore, we combined the two size classes when we calculated caterpillar density. The central subpopulation had a 4437% (45×) lower density of leaf‐roller caterpillars (site mean ± SE caterpillars/ha = 14.23 ± 7.02; log‐transformed = 1.36 ± 0.57) than the eastern subpopulation (site mean ± SE caterpillars/ha = 645.65 ± 387.82; log‐transformed = 4.20 ± 0.98; *t =* −2.49, *df =* 18, *p =* .02; Figure 2a). In contrast, smooth caterpillar density was 423% (5×) greater in the central subpopulation (site mean ± SE caterpillars/ha = 3334.54 ± 906.03; log‐transformed = 7.77 ± 0.28) than the eastern subpopulation (site mean ± SE caterpillars/ha = 636.45 ± 143.82; log‐transformed = 6.19 ± 0.25; *t =* 4.16, *df =* 18, *p =* <.01; Figure 2a).

**FIGURE A3:**
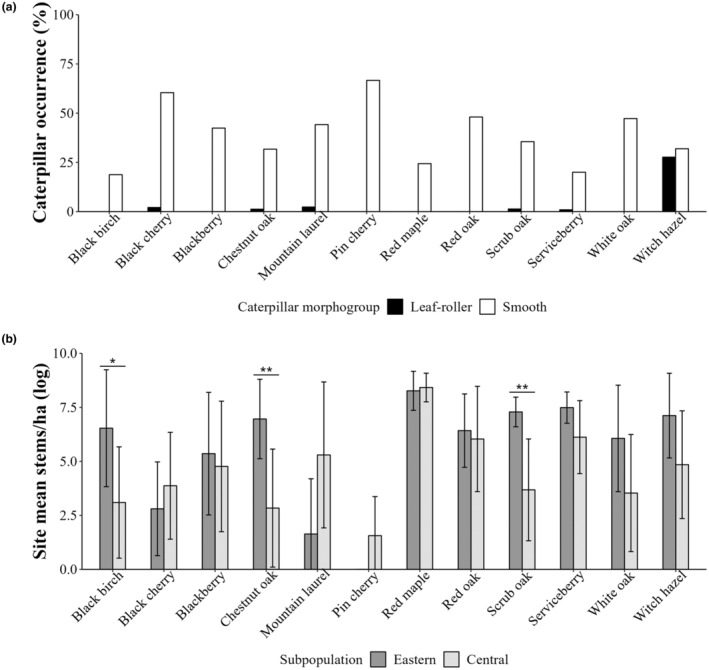
Caterpillars and 12 focal species were sampled at 10 sites in each subpopulation in Pennsylvania. Comparison across the focal species for caterpillar percent occurrence (a). Caterpillar percent occurrence is defined as how often each morphogroup (leaf‐roller and smooth) was present when a focal plant was searched. Comparison among the 12 sapling and shrub focal species between the subpopulations (log‐transformed; b). Statistical differences from two‐tailed *t*‐tests are indicated with *p ≤ .05 and **p ≤ .01. Error bars represent 95% confidence intervals.

Across all sites, leaf‐roller caterpillars occurred predominantly on witch hazel (28% of caterpillar occurrences) and the percent occurrence of leaf‐roller caterpillars on witch hazel was 8% and 43% in the central and eastern subpopulations, respectively (Figure A3a). Smooth caterpillars occurred predominantly on pin cherry (67% of caterpillar occurrences) and the percent occurrence of smooth caterpillars on pin cherry was 69% and 50% in the central and eastern subpopulations, respectively (Figure A3a). The eastern subpopulation had greater densities of black birch (*t* = 2.08, *df* = 18, *p* = .05), chestnut oak (*t* = 2.83, *df* = 18, *p* = .01), and scrub oak (*t* = 3.32, *df* = 18, *p* < .01; Figure A3b). The average plant area of the 12 pooled focal species searched for caterpillars (*t =* 0.02, *df =* 904, *p =* .98) and average plant area of witch hazel (*t =* −0.35, *df =* 92, *p =* .73) were similar between subpopulations.

### 3.2 Plasma metabolites and adult body mass

During 4 May–7 June 2018, we collected a total of 111 blood samples (central: 59 and eastern: 52) from adult male Golden‐winged Warblers within 22 managed nesting sites. We performed plasma lipid metabolite assays on 82 plasma samples (central: 42 and eastern: 40; 29 samples were excluded for having too little plasma volume or hemolyzed plasma; Table A1). Of these 82 plasma samples, six male adult warblers were sampled during both visits (central: 2 and eastern: 4). Therefore, we collected plasma samples from 76 unique adult male warblers. The average sample coefficient of variation for the 43 samples run in duplicates were 16% and 6% for TRIG and GLYC, respectively.

From our backwards stepwise selection process to test our prediction that plasma lipid metabolites and adult male body mass were influenced by the interaction and main effects of subpopulation and breeding stage, we found that our final TRIG model included the interaction term (subpopulation × breeding stage) and SMI (*F* = 2.48, *df* = 4 and 77, *p* = .05, *R* ^2^ = .11, *adjusted R* ^2^ = .07). The *β* 90% CIs did not overlap zero for the interaction term (*β* estimate = 0.95 and 0.23–1.67), the two main effects (subpopulation: *β* estimate = −0.69 and −1.23 to −0.14 and breeding stage: *β* estimate = −0.63 and −1.15 to −0.11), nor SMI (*β* estimate = 0.22 and 0.03–0.41). TRIG concentrations peaked during the breeding‐grounds‐arrival stage in the eastern subpopulation but were similar between subpopulations during the nestling‐rearing stage (Figure 2b). The change in TRIG concentrations from the breeding‐grounds‐arrival stage to the nestling‐rearing stage depended on subpopulation; whereby, concentrations did not change in the central subpopulation and concentrations decreased in the eastern subpopulation (Figure 2b; Table A1). We found that our final GLYC model included the subpopulation main effect and SMI (*F* = 7.22, *df* = 2 and 79, *p* < .01, *R* ^2^ = .15, *adjusted R* ^2^ = .13). The *β* 90% CIs did not overlap zero for the main effect (subpopulation: *β* estimate = −0.53 and −0.89 to −0.16), nor SMI (*β* estimate = 0.22 and 0.04–0.40). Although in the eastern subpopulation GLYC tended to increase from the first‐arrival stage to the nestling‐rearing stage and there was no change in the central subpopulation, this interaction was not strong enough and was removed (interaction only included for graphing purposes in Figure 2c; Table A1). Our final adult male body mass model included the subpopulation main effect and wing chord (*F* = 6.70, *df* = 2 and 79, *p* < .01, *R* ^2^ = .15, *adjusted R* ^2^ = .12). The *β* 90% CIs did not overlap zero for the main effect (subpopulation: *β* estimate = −0.63 and −0.97 to −0.28), nor wing chord (*β* estimate = 0.18 and 0.01–0.36). Similar to our GLYC results, although in the eastern subpopulation adult male body mass tended to increase from the first‐arrival stage to the nestling‐rearing stage and there was no change in the central subpopulation, this interaction was not strong enough and was removed (interaction only included for graphing purposes in Figure 2d; Table A1).

### 3.3 Fledgling body mass

During field work from McNeil et al. (2024), 156 Golden‐winged Warbler fledglings were weighed from 19 managed nesting sites (central: 66 and eastern: 90) and 24 (15%, 24/156) were opportunistically weighed (central: 12 and eastern: 12). We weighed 80 individuals before fledging (central: *n* = 15, median = 9 days, min = 7 days, max = 9 days and eastern: *n* = 65, median = 8 days, min = 6 days, max = 8 days) and 76 individuals after fledging (central: *n* = 51, median = 9 days, min = 9 days, max = 17 days and eastern n = 25, median = 9 days, min = 8 days, max = 22 days). Before fledging, central subpopulation fledglings were 16% lighter (mean = 7.58, 95% CI: 7.36–7.81) than the eastern subpopulation (mean = 8.30, 95% CI: 8.20–8.41; *t* = −5.78, *df* = 78, *p* < .01; Figure 2e). Additionally, after fledging, central subpopulation fledglings were 16% lighter (mean = 7.62, 95% CI: 7.36–7.88) than the eastern subpopulation (mean = 8.86, 95% CI: 8.49–9.23; *t* = −5.53, *df* = 74, *p* < .01; Figure 2e). Fledgling body mass did not vary from 2014 to 15 in the eastern subpopulation for both age groups (before: *t* = 0.04, *df* = 63, *p* = .97 and after: *t* = 1.60, *df* = 23, *p* = .12) nor from 2016 to 17 in the central subpopulation for both age groups (before: *t* = 0.31, *df* = 13, *p* = .76 and after: *t* = 0.45, *df* = 49, *p* = .66).

## 4 DISCUSSION

Following recent work that documented Golden‐winged Warblers in Pennsylvania's central subpopulation exhibit poor breeding productivity (McNeil et al., 2024; McNeil, Rodewald, Robinson, et al., 2020) and struggle to respond to regional conservation efforts (McNeil, Rodewald, Ruiz‐Gutierrez, et al., 2020), we tested our hypothesis that the central subpopulation experienced lower food availability. We found support for our primary prey density (leaf‐roller caterpillars) and fledgling body mass predictions, but we found mixed support for our plasma lipid metabolites and adult male body mass predictions. Although our findings did not align with all our predictions, we believe that the lines of evidence we present (leaf‐roller caterpillar density, plasma lipid metabolites, adult and fledgling body mass) support the hypothesis that the central subpopulation experiences lower food availability and, by extension, less available food is likely driving the disparity in breeding productivity rates and subsequent responses to conservation (McNeil et al., 2024; McNeil, Rodewald, Robinson, et al., 2020; McNeil, Rodewald, Ruiz‐Gutierrez, et al., 2020). When comparing full‐season productivity from our two study subpopulations (eastern: 3.07 and central: 1.08 [McNeil, Rodewald, Robinson, et al., 2020]) to other regions of the warblers' Appalachian breeding range, this metric was found to be even lower in Tennessee (0.66 [Lehman, 2017]), a state also experiencing steep population declines (1966–2021 −7.2%/year^−1^ [Sauer et al., 2021]). As such, future work should attempt to evaluate food availability and its potential to limit reproductive success, especially across other Appalachian subpopulations of Golden‐winged Warblers. Collectively, our results contribute to a growing body of evidence suggesting that conservation outcomes for a migratory bird can be facilitated—or stifled—by insect prey abundance (Grames et al., 2023; Martay et al., 2023; Møller, 2019).

We predicted that the central subpopulation would have fewer leaf‐roller caterpillars due to lower reported warbler breeding productivity rates in this region (McNeil, Rodewald, Robinson, et al., 2020). Although we found smooth caterpillar density was 423% (5×) greater in the central subpopulation than the eastern subpopulation, leaf‐roller caterpillar density, the morphogroup upon which Golden‐winged Warblers are known to heavily specialize (Streby et al., 2014), was 4437% (45×) fewer in the central subpopulation than the eastern subpopulation. Indeed, Golden‐winged Warblers forage with a specialized probe‐and‐gape behavior that allows them to extract insects (e.g., leaf‐roller caterpillars) from curled leaves (Chandler et al., 2016; Confer et al., 2020). Even though we found that smooth caterpillar density was higher in the central subpopulation, this likely does not compensate for fewer leaf‐rollers given that many other insectivorous birds are known to compete with Golden‐winged Warblers for the former morphogroup (Holmes & Schultz, 1988; Jones et al., 2016; Robinson & Holmes, 1982). Reduced caterpillar abundance has been found to drive population demographics in other warbler species as well; such as, lower likelihood of double brooding by Black‐throated Blue Warblers (*Dendroica caerulescens* [Nagy & Holmes, 2005]) and reduced breeding productivity by Worm‐eating Warblers (*Helmitheros vermivorum* [Awkerman et al., 2011]). Our finding of fewer leaf‐roller caterpillars in the central subpopulation is in alignment with multiple other reported metrics indicative of poor reproductive success in this region; such as, smaller clutch sizes, lower nestling body mass, lower nestling survival rates, lower fledgling survival rates, lower full‐season productivity, and greater begging by fledglings (McNeil et al., 2024; McNeil, Rodewald, Robinson, et al., 2020) and our findings of lower adult male and fledgling body mass. Lower parental provisioning due to poorer habitat is an early‐life stressor that can affect nestlings' glucocorticoid responses (i.e., elevate glucocorticoids [as in Kitaysky et al., 2010]), and could conceivably affect stress responses and condition when those nestlings become adults (Romero, 2004). Our findings and other work (McNeil et al., 2024; McNeil, Rodewald, Robinson, et al., 2020) suggest Golden‐winged Warblers in the central subpopulation are less successful than those in the eastern subpopulation, but further study is needed to understand how prey limitation at any life stage may carry‐over to affect birds in this subpopulation later.

As predicted, upon breeding grounds arrival, TRIG concentrations were lower in the central subpopulation, which suggests these birds deposited less fat than those in the eastern subpopulation during this time. Although higher rates of fat deposition in the eastern subpopulation during breeding grounds arrival compliments our finding of greater leaf‐roller caterpillar densities, the inferences we can make by linking food availability to our TRIG concentrations during this stage are limited given that we sampled caterpillars (30 May–15 June) closer to when adults provisioned their young (5–15 June) rather than breeding grounds arrival (4–9 May). In fact, when we found leaf‐roller caterpillars to be 45× lower in the central subpopulation TRIG concentrations were the same (nestling‐rearing stage). But we believe other factors are driving this pattern during the nestling‐rearing stage (discussed in next paragraph). Although we did not sample caterpillars and collect blood samples at the same time, caterpillar abundance and composition were found to fluctuate significantly over the course of a single growing season in Virginia (Seifert et al., 2021). Despite this, leaf‐rollers (Tortricidae) were found to be the most abundant family during this study's early‐season‐sampling period (26 April–6 June [Seifert et al., 2021]) which overlaps with our Golden‐winged Warbler breeding‐grounds‐arrival stage. If the trends observed later in the season hold true for earlier in the season, then we would expect had we also sampled caterpillars when Golden‐winged Warblers arrived at their breeding grounds we may have documented an even greater difference in leaf‐roller caterpillar density between the two subpopulations.

Our predictions of lower TRIG concentrations during the nestling‐rearing stage and higher GLYC concentrations across both breeding stages in the central subpopulation were not supported by our results. We found that the change in TRIG concentrations from the breeding‐grounds‐arrival stage to the nestling‐rearing stage was subpopulation dependent (eastern: decrease, central: no change) which may suggest the interaction was driven by energy demand differences in the two subpopulations. Indeed, other studies that have measured plasma lipid metabolites for adult male songbirds during the nestling‐rearing stage and identify energy demands as a driver for their metabolite findings (Done et al., 2011; Kern et al., 2005; Owen et al., 2005). Our eastern subpopulation is more dense (i.e., greater territory defense demands [Larkin & Bakermans, 2012; McNeil, 2015; McNeil, Rodewald, Ruiz‐Gutierrez, et al., 2020]) and reproductive success is greater (i.e., greater provisioning demands [McNeil, Rodewald, Robinson, et al., 2020]) compared to the central subpopulation. Songbirds are known to lose mass and fat stores during the breeding season due to energetic costs of reproduction (Witter & Cuthill, 1993) and our TRIG findings reflect the known differences in reproductive success between the two subpopulations (eastern: high and central: low [McNeil, Rodewald, Robinson, et al., 2020]); whereby, the eastern subpopulation males experienced higher reproductive cost (decrease in TRIG) and the central subpopulation males experienced lower reproductive cost (no change in TRIG). Although we believe that subpopulation energy demand differences were the driver of our TRIG interaction result, TRIG concentrations were still similar between subpopulations during the nestling‐rearing stage. This may be a product of leaf‐roller caterpillar density being 45× denser in the eastern subpopulation during this time; therefore, more available food alleviated some of the energy costs of reproduction. This pattern of greater energy demands in the eastern subpopulation is further supported by our finding that GLYC concentrations were higher in this region across both breeding stages. One study documented male Pied Flycatchers (*Ficedula hypoleuca*) to have elevated concentrations of other metabolites indicative of fat catabolism during the nestling‐rearing stage and similarly suggested this pattern to likely be driven by territory defense and nestling provisioning (Kern et al., 2005). Indeed, male Golden‐winged Warblers provision nestlings as much or more than females (Reed et al., 2007).

Lastly, we found that adult male warblers in the central subpopulation had lower body mass across both breeding stages and we did not find body mass to be similar upon breeding grounds arrival as we predicted. Upon breeding grounds arrival, we found that the average adult male warbler body mass in the central subpopulation was 35% less than the eastern subpopulation. This disparity in adult male body mass upon arrival could be driven by a combination of multiple factors. Given that breeding Golden‐winged Warblers from the central and eastern subpopulation overwinter together (thus suggesting non‐breeding grounds are not responsible; Kramer et al., 2018), one reasonable hypothesis would be a carry‐over effect from birds' poor condition during their own nestling/fledgling stages (Akresh et al., 2019; Mainwaring et al., 2023). Although the natal philopatry for Golden‐winged Warblers is largely unknown, it is believed that individuals from these two subpopulations do not mix on the breeding grounds given the extent of unoccupied habitat between these two regions (McNeil, Rodewald, Ruiz‐Gutierrez, et al., 2020). Alternatively, the eastern subpopulation, which is settled sooner by breeding males than the central subpopulation (McNeil, 2019), may have heavier males because it is well documented that birds of better body condition arrive at higher‐quality breeding sites first (Kokko, 1999). Importantly, although we found warblers from the eastern subpopulation catabolized more fat across both breeding stages, birds in this region still maintained a higher body mass. Change in body mass from the breeding‐grounds‐arrival stage to the nestling‐rearing stage was 0.15 g more in the eastern subpopulation than the central subpopulation. This finding further supports our hypothesis that the lower available food may affect demographic rates in the central subpopulation, as greater body mass has been reported to be an indicator of superior fitness in other species (e.g., Ovenbird [*Seiurus aurocapilla*; Mazerolle & Hobson, 2002]).

**FIGURE A4:**
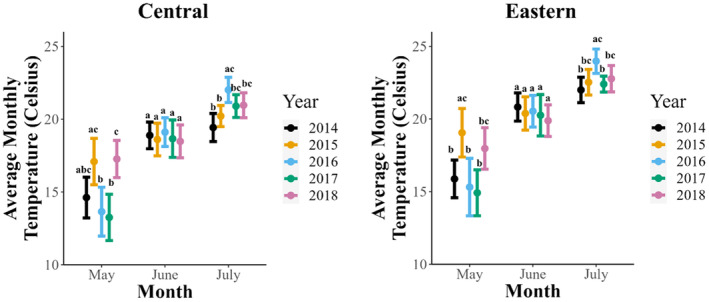
Average monthly temperatures (°C) across the five sampling years were compared separately within each subpopulation and each month (6 separate comparisons). We acquired average daily temperature from the closest weather station to each subpopulation (central: University Park and eastern: Wilkes Barre Scranton International Airport) from NOAA's Local Climatological Data (www.ncdc.noaa.gov/cdo‐wed/datatools/lcd). We used an ANOVA test followed by Tukey HSD for our comparisons. Lower case letters denote differences in average temperature (*p* < .05).

Although we present several lines of evidence in support of our food‐availability hypothesis, it is important to consider our study's limitations. First, we employed a rapid caterpillar sampling method that did not truly standardize caterpillar abundance to leaf area or caterpillar size (Cooper & Whitmore, 1990) and we did not sample caterpillars from vegetation >3.5 m (i.e., excluded canopy trees) which other male warbler species are known to preferentially forage at heights near their song perches (Holmes, 1986). Despite not standardizing for leaf area, we found that average witch hazel plant area was similar between subpopulations which may have controlled for some variation in caterpillar abundance and leaf area. As described in our methods, our smooth caterpillar morphogroup may have included leaf‐roller (Tortricidae) and non‐leaf‐roller species; therefore, the relative difference between our leaf‐roller and smooth caterpillar densities may be lower than we report. Secondly, the three datasets we analyzed were not synchronized in time. Consequently, were there a marked “year” effect, it could potentially produce the patterns we observed. Thus, future work that measures prey availability, nest survival, fledgling survival/behavior, and plasma lipid metabolites all during the same time period would be valuable. To evaluate how comparable our five sampling (2014–18) years were, we compared average monthly temperature across years within each subpopulation (Figure A4). Most importantly, we found that average June temperatures did not vary across the 5 years and this month was when we conducted most of the caterpillar sampling. Indeed, caterpillar abundance in eastern deciduous forests have been found to vary relatively little among years with similar temperatures (Reynolds et al., 2007). Lastly, due to the challenges of capturing females with conspecific playback methods, we did not sample blood and body mass from adult females which excludes an important component given other songbird species are known to exhibit sex‐specific differences in physiological conditions during the breeding season (Done et al., 2011; Kern et al., 2005; Owen et al., 2005).

Collectively, our results suggest that poor breeding success of Golden‐winged Warblers in the central subpopulation could be driven by lower availability of primary prey (leaf‐roller caterpillars) during the breeding season, and this, in turn, limits their response to conservation efforts. Our findings support that management practices focused on supporting abundant food resources (woody host plants for caterpillars) should be a focus within Golden‐winged Warbler nesting habitat best management practices (Bellush et al., 2016; Roth et al., 2019). Oaks (*Quercus* spp.), blackberry (*Rubus* spp.), and cherries (*Prunus* spp.) have been found to host particularly high diversity of lepidoptera larvae (Narango et al., 2020) and these genera were also found to be selectively foraged on by Golden‐winged Warblers in our central subpopulation (Bellush et al., 2016). Because 94% of leaf‐roller caterpillars we sampled were on witch hazel and this was found to be one of 10 indicator species for sites occupied by Golden‐winged Warblers in a previous study within our central subpopulation (Bellush, 2012), witch hazel may be a particularly valuable resource to retain with managed nesting sites. Management recommendations for this imperiled songbird would be further informed by future studies focused on elucidating the drivers behind differential leaf‐roller caterpillar density between the two subpopulations. Although not analyzed here, potential drivers that we believe are worth exploring include impacts from climate change (Harvey et al., 2023), differences in wetland abundance or forest‐community type (e.g., natural wetlands are more abundant in the eastern subpopulation [Davis, 1993]), and spongy moth (*Lymantria dispar*) competition and/or control (Awkerman et al., 2011; Leroy et al., 2021; Marshall et al., 2002; Timms & Smith, 2011). Furthermore, fecal metabarcoding could be used to advance our understanding of Golden‐winged Warblers' diet (Verkuil et al., 2022).

## AUTHOR CONTRIBUTIONS

**Emma C. Keele:** Data curation (equal); formal analysis (lead); project administration (supporting); visualization (lead); writing – original draft (lead). **Cameron J. Fiss:** Conceptualization (equal); data curation (equal); formal analysis (supporting); investigation (equal); methodology (equal); project administration (equal); supervision (equal); validation (equal); visualization (supporting); writing – original draft (supporting); writing – review and editing (equal). **Darin J. McNeil:** Conceptualization (equal); data curation (equal); formal analysis (supporting); investigation (equal); methodology (equal); project administration (equal); supervision (equal); validation (equal); visualization (supporting); writing – original draft (supporting); writing – review and editing (equal). **Meredith Anderson:** Formal analysis (supporting); validation (supporting); visualization (supporting); writing – original draft (supporting); writing – review and editing (equal). **Nathan Thomas:** Formal analysis (supporting); validation (supporting); writing – review and editing (supporting). **Dakotah Shaffer:** Data curation (equal); investigation (supporting); writing – review and editing (supporting). **Jeffery L. Larkin:** Conceptualization (equal); funding acquisition (lead); investigation (supporting); methodology (equal); project administration (equal); supervision (equal); validation (equal); writing – original draft (supporting); writing – review and editing (equal).

## CONFLICT OF INTEREST STATEMENT

The authors declare no conflicts of interest associated with this manuscript.

## Data Availability

Our dataset and R code are available in Dryad and can be accessed with the following link: https://doi.org/10.5061/dryad.z08kprrmm.

## APPENDIX A

**TABLE A1:** Mean (non‐transformed) triglyceride and glycerol concentrations and body mass (and 95% confidence intervals) measured from adult male Golden‐winged Warblers in eastern and central subpopulations in Pennsylvania.

Variable	Breeding stage	Eastern	Central
Triglycerides (mM)	First‐arrival	1.10 (0.88, 1.32)	0.81 (0.67, 0.95)
	Nestling‐rearing	0.92 (0.73, 1.00)	0.99 (0.79, 1.05)
Glycerol (mM)	First‐arrival	0.84 (0.72, 0.97)	0.77 (0.69, 0.85)
	Nestling‐rearing	0.94 (0.86, 1.02)	0.77 (0.71, 0.83)
Adult body mass (g)	First‐arrival	9.33 (9.02, 9.64)	9.05 (8.78, 9.32)
	Nestling‐rearing	9.50 (9.32, 9.68)	9.07 (8.83, 9.31)

*Note*: Sampling occurred during two breeding stages, breeding grounds arrival (First‐arrival: 4–9 May) and peak adult rearing of nestlings (Nestling‐rearing: 5–12 June). Data were analyzed for 76 adult male warblers, six of which were recaptured (82 total plasma samples: Eastern/First‐arrival = 17, Central/First‐arrival = 19, Eastern/Nestling‐rearing = 23, Central/Nestling‐rearing = 23).
